# ABO Blood Type and Urinary Bladder Cancer: Phenotype, Genotype, Allelic Association with a Clinical or Histological Stage and Recurrence Rate

**DOI:** 10.1055/s-0044-1788614

**Published:** 2024-07-22

**Authors:** Ivan Milas, Željko Kaštelan, Jószef Petrik, Jasna Bingulac-Popović, Bojan Čikić, Andrej Šribar, Irena Jukić

**Affiliations:** 1 Department of Urology, University Hospital Centre Zagreb, Zagreb, Croatia; 2 Department of Medical Sciences, Croatian Academy of Sciences and Art, Zagreb, Croatia; 3 Department of Medical Biochemistry and Hematology, Faculty of Pharmacy and Biochemistry, University of Zagreb, Zagreb, Croatia; 4 Medical Department, Croatian Institute of Transfusion Medicine, Zagreb, Croatia; 5 Clinical Department of Anesthesiology and Intensive Care Medicine, Dubrava University Hospital , Zagreb, Croatia; 6 Faculty of Medicine, Josip Juraj Strossmayer University of Osijek, Osijek, Coratia

**Keywords:** ABO blood groups, urinary bladder cancer, ABO phenotype, ABO genotype, ABO allele, case–control study

## Abstract

**Background** Previous research on connection between the ABO blood group and bladder cancer has been based on determining the ABO phenotype. This specific research is extended to the molecular level, providing more information about particular ABO alleles.

**Aim** To investigate the impact of the ABO blood group genotype or phenotype as a risk factor for urinary bladder cancer.

**Materials and Methods** In the case–control study, we included 74 patients who underwent surgery for a urinary bladder tumor at the Urology Clinic, Clinical Hospital Centre Zagreb, in 2021 and 2022. The control group comprised 142 asymptomatic and healthy blood donors. ABO genotyping to five basic alleles was done using a polymerase chain reaction with sequence-specific primers. We compared ABO phenotypes, genotypes, and alleles between patients and the healthy controls and investigated their distribution according to the clinical and histological stage and recurrence rate.

**Results** No statistically significant difference was found among the groups, nor for the observed disease stages in terms of the phenotype and genotype. At the allele level, the results show a significantly lower proportion of malignancy in O1 ( *p* < 0.001), A1 ( *p* < 0.001), and B ( *p* = 0.013), and a lower proportion of metastatic disease in A2 (0%, *p* = 0.024). We also found significantly higher proportions of high-grade tumors in patients with O1 (71.4%, *p* < 0.001), A1 (70.1%, *p* = 0.019), of nonmuscle invasive tumors in patients with O1 (55.1%, *p* < 0.001), O2 (100%, *p* = 0.045), and recurrent tumors in patients with O1 (70.2%, *p* < 0.001) and A1 (74.2%, *p* = 0.007) alleles.

**Conclusion** We did not find an association between the ABO blood group genotype or phenotype as a genetic risk factor for urinary bladder cancer. However, an analysis at the allelic level revealed a statistically significant association between certain alleles of the ABO blood group system and urinary bladder tumors, clinical or histological stage, and recurrence rate, respectively.

## Introduction

Tumors treated in urology as a clinical branch of medicine cover a wide range of mostly malignant diseases within the human urinary and male reproductive systems. Tumors affecting the urinary system can be found in the adrenal gland, kidney, ureter, bladder, and urethra, and in the male reproductive system, such as tumors of the testicles, prostate, and penis. Management of genitourinary malignancy is likely to demand a large portion of the urologist's time and practice, and the challenge for modern urology is not only in understanding the management of commonly seen genitourinary malignancies but also in identifying significant associations that can be risk factors in the development of malignant tumors. 1 Many biomarkers have been studied as assessors of outcomes in the development and treatment of malignant diseases, and one of them is the ABO blood group. Despite a multitude of studies attempting to correlate the ABO phenotype with cancer risk, the link between the expression of histoblood group antigens and tumorigenesis has remained unclear for most evaluated tumor types. 2

Bladder cancer (BC) is ranked tenth among the most commonly diagnosed cancers for both genders and appears more often in men. 3 Histologically, in 90% of cases, it involves urothelial carcinoma. 4 The most common symptom that indicates the presence of a bladder tumor is painless macrohematuria. In some cases, they are asymptomatic and are detected when diagnosing other diseases. Several risk factors associated with BC have been identified, and the main risk is active and passive tobacco smoking. 5 The main diagnostic and therapeutic procedure of BC is cystoscopy and transurethral resection (TUR) or cold punch biopsy of the tumor. 6 Tumor staging with multislice computed tomography and histopathological evaluation are used to guide treatment and determine a prognosis for BC. 7 The important clinical approach is to confirm whether it is a superficial (nonmuscle invasive, NMI), muscle-invasive (MI), or metastatic (M) disease. Another important factor in predicting risks of disease progression in NMI tumors is the tumor grade and recurrence rate. 8 Traditionally, radical cystectomy is recommended in MI tumors without distant metastases, an extensive papillary disease that cannot be controlled with TUR, or in M disease together with systemic chemotherapy as a salvage procedure in preventing blood loss (hematuria). 9

Thanks to Karl Landsteiner, the ABO blood type system was identified in the early 20th century using serological methods that have remained the standard. 10 As new technologies have refined the ability to identify genome sequences, a single gene on chromosome 9q34 was finally found to define a person's blood group and its nucleotide sequence was elucidated in the year 1990. 11 12 Today, we know that the gene that determines the blood type encodes a glycosyltransferase with three main allelic forms: A, B, and O. 13 The A, B, and O glycosyltransferases transfer N-acetylgalactosamine, D-galactose, and no sugar residue, to a protein known as H antigen, which is expressed on the surface of red blood cells and other tissues through the body. 14

Since 1999, the BGMUT (Blood Group antigen gene MUTation) Database has been documenting genes of human blood group systems using single-nucleotide polymorphisms (SNPs). 15 SNP or RS represents a single base substitution present at a specific location of the gene, which we can use to identify a specific gene region. The advantages of using SNPs in genotyping are their abundance in genomes, stability at a low mutation rate, and short fragment amplification. 16 For the purpose of ABO blood group genotyping, the following sequences of SNPs have been identified: rs 507666 as the perfect surrogate for type A1, rs687289 as a marker for the O allele, rs8176746 for the B allele, and rs8176704 for the A2 allele according to BGMUT Database ( www.ncbi.nlm.nih.gov ). Today, DNA-based genotyping is used as an alternative to the serological antibody-based method to determine a person's blood group, such as in patients with multiple antigens in blood sample, in patients with recent transfusion treatment (masked antigens), and in obstetrics for identifying D phenotype due to use of anti-D immunoglobulin. 17

### Aim

The aim of our research is to evaluate the association between the ABO blood group genotype/phenotype and the risk for urinary BC. In addition, we examined the association between the ABO blood group and tumor grade, clinical stage of disease (NMI, MI, M), and incidence of disease recurrence in NMI tumors.

## Materials and Methods

### Study Participants

The study group comprised 74 patients who underwent surgery for a urinary bladder tumor at the Urology Clinic, University Clinical Hospital Centre Zagreb, in 2021 or 2022. The study received approval from the Ethics Committee of the University Clinical Hospital Center Zagreb number: 02/21 AG from November 2, 2020, and all procedures were performed in compliance with relevant laws and institutional guidelines. Preoperative blood samples were taken from the patients who had given signed consent. Laboratory analysis of the ABO blood group genotype was analyzed by the Department of Molecular Diagnostics at the Croatian Institute of Transfusion Medicine. The control group comprised 142 asymptomatic and healthy (female and male) blood donors without a medical history of tumors. The median age of the participants in the control group was 51 years. All participants in the control group resided in the same geographic region.

A TUR or radical cystectomy was performed in patients with a urinary bladder tumor. 18 19 Specimens were processed according to standard histopathological procedures and evaluated by experienced uropathologists in a high-volume center. The tumors were histologically graded as high grade (HG) and low grade 20 and then divided into three groups: NMI (superficial), MI, and M tumors. The clinical stage of the disease was tagged according to the Tumor Node Metastasis classification system. 21

### ABO Genotyping

Genomic DNA isolation from EDTA blood samples was performed using the commercial QIAmp DNA Mini kit (Qiagen, Hilden, Germany). ABO genotyping to given basic ABO alleles (O1, O2, A1, A2, B) and 15 genotypes was done by using the sequence-specific primer (SSP) method (polymerase chain reaction [PCR] with SSPs) in 8 parallel PCR-SSP reactions to amplify exons 6 and 7 of the ABO blood group gene. 22

The presence of a gene fragment of the human growth hormone (as an internal control) in each SSP reaction showed that amplification was successful. The ABO alleles and genotypes were named according to the nomenclature used by Yamamoto. 23

### Statistical Analysis

The data are presented in tables. Continuous variables are displayed as either mean or standard deviation for values with a Gaussian distribution or median and interquartile range (IQR) for data that does not follow the normal distribution. The normality of the distribution was assessed using the Shapiro–Wilk test. Categorical variables are displayed as counts and percentages.

Differences in independent continuous variables between the two groups were tested for statistical significance using the Student's *t* -test for independent samples or the Wilcoxon rank-sum (Mann–Whitney U) test, depending on the distribution of data.

Differences in categorical variables were tested for statistical significance using the Pearson's χ ^2^ test.

*p* -Values < 0.05 were considered statistically significant, and the software package used for statistical analysis was jamovi v2.3.

## Results

### Healthy Controls

There were 142 healthy controls over 40 years of age, 96 males and 46 females. The median age was 51 years (IQR: 46–57). According to the blood type phenotypes, 52 (36.6%) were blood type O, 62 (43.7%) were type A, 22 (15.5%) were type B, and 6 (4.2%) were type AB. According to the genotypes, 48 (33.8%) were O1A1, 47 (33.1%) were O1O1, 21 (14.8%) were O1B, 6 (4.2%) were A1B, 5 (3.5%) were of O1O2, O1A2, and A1A1 genotype, 2 (1.4%) were O2A1 and A1A2, and 1 patient (0.7%) was of BB genotype ( Table 1 ).

**Table 1:** Comparison of ABO phenotypes and genotypes between healthy controls and patients with bladder cancer, χ ^2^ test, *p* = 0.948, 0.347

ABO phenotype	Bladder cancer, *n* (%)	Control group, *n* (%)	ABO genotype	Bladder cancer, *n* (%)	Control group, *n* (%)
O			O1O1	24 (34.3)	47 (33.1)
	25 (35.8)	52 (36.6)	O1O2	1 (1.4)	5 (3.5)
			O2O2		
A			A1A1	4 (5.7)	5 (3.5)
			O1A1	19 (27.1)	48 (33.8)
	33 (47.2)	62 (43.7)	O2A1	0	2 (1.4)
			A1A2	2 (2.9)	2 (1.4)
			O1A2	5 (7.1)	5 (3.5)
			O2A2		
			A2A2	2 (2.9)	0
B			BB	0	1 (0.7)
	9 (12.6)	22 (15.5)	O1B	7 (10)	21 (14.8)
			O2B	1 (1.4)	0
AB	3 (4.3)	6 (4.2)	A1B	4 (5.7)	6 (4.2)
			A2B	1 (1.4)	0
Total	70 (99.9)	142 (100.0)		70 (100.0)	142 (100.0)

### Bladder Cancer

There were 74 patients with BC in the cohort, of which 62 were males (83.8%), significantly more compared with the healthy controls (83.8 vs. 59.4%, *p* < 0.001). There was incomplete data on four patients, and they were excluded from further analyses. There was no statistically significant difference between the groups regarding the ABO phenotype ( *p* = 0.948) or genotype ( *p* = 0.347) distribution ( Table 1 ).

In patients with cancer, there was no significant association between histological grades, invasiveness or the occurrence of relapse and phenotypes, grade ( *p* = 0.710), invasiveness ( *p* = 0.210), relapse ( *p* = 0.145, Table 2 ), or genotypes, grade ( *p* = 0.529), invasiveness ( *p* = 0.830), relapse ( *p* = 0.747, Table 3 ).

**Table 2:** Comparison of ABO phenotypes between histological grades, invasiveness, and occurrence of relapse

Phenotype	Grade		Invasiveness			Relapse	
	HG (%)	LG (%)	M (%)	MI (%)	NMI (%)	No (%)	Yes (%)
O	17 (68)	8 (32)	5 (20)	5 (20)	15 (60)	18 (72)	7 (28)
A	23 (69.7)	10 (30.3)	4 (12.1)	14 (42.4)	15 (45.5)	26 (78.8)	7 (21.2)
B	6 (66.7)	3 (33.3)	2 (22.2)	1 (11.1)	6 (66.7)	4 (44.4)	5 (55.6)
AB	3 (100)	0 (0)	1 (33.3)	2 (66.7)	0 (0)	3 (100)	0 (0)
Total	49 (70)	21 (30)	12 (17.1)	22 (31.4)	36 (51.4)	51 (72.9)	19 (27.1)

Abbreviations: HG, high grade; LG, low grade; M, metastatic; MI, muscle invasive; NMI, nonmuscle invasive.

Note: χ ^2^ test, grade *p* = 0.710, invasiveness *p* = 0.210, relapse *p* = 0.145.

**Table 3:** Comparison of ABO genotypes between histological grades, invasiveness, and occurrence of relapse

Genotype	Grade		Invasiveness			Relapse	
	HG (%)	LG (%)	M (%)	MI (%)	NMI (%)	No (%)	Yes (%)
O1O1	16 (66.7)	8 (31.6)	5 (21.7)	5 (21.7)	13 (56.5)	17 (70.8)	7 (29.2)
O1A1	13 (68.4)	6 (31.6)	3 (15.8)	7 (36.8)	9 (47.4)	15 (78.9)	4 (21.1)
O1A2	4 (80)	1 (20)	0 (0)	3(60)	2 (40)	4 (80)	1 (20)
O1B	6 (85.7)	1 (14.3)	2 (28.6)	1 (14.3)	4 (57.1)	4 (57.1)	3 (42.9)
A1A1	3 (75)	1 (25)	1 (25)	1 (25)	2 (50)	3 (75)	1 (25)
O1O2	1 (100)	0 (0)	0 (0)	0 (0)	1 (100)	1 (100)	0 (0)
A1A2	2 (100)	0 (0)	0 (0)	2 (100)	0 (0)	2 (100)	0 (0)
A1B	3 (75)	1 (25)	1 (25)	2 (50)	1 (25)	3 (75)	1 (25)
A2B	0 (0)	1 (100)	0 (0)	0 (0)	1 (100)	0 (0)	1 (100)
A2A2	1 (100)	0 (0)	0 (0)	1 (100)	0 (0)	1 (100)	0 (0)
O2B	0 (0)	1 (100)	0 (0)	0 (0)	1 (100)	0 (0)	1 (100)
O1A2	0 (0)	1 (100)	0 (0)	0 (0)	1 (100)	1 (100)	0 (0)
Total	49 (70)	21 (30)	12 (17.1)	22 (31.4)	36 (51.4)	51 (27.1)	19 (72.9)

Abbreviations: HG, high grade; LG, low grade; M, metastatic; MI, muscle invasive; NMI, nonmuscle invasive.

Note: χ ^2^ test, grade *p* = 0.529, invasiveness *p* = 0.830, relapse *p* = 0.747.

### Alleles

While there is a higher incidence of malignant disease in patients with the A2 allele (58.8%) compared with O1 (30.8%), O2 (30%), A1 (31,3%), and B (30.9%), no significant difference was found (χ ^2^ = 5.84, *p* = 0.211). However, these results are still underpowered (1 − β = 0.72), and a larger sample is required to confirm the null hypothesis that there is no association between alleles and the incidence of malignant disease.

**Fig. 1:**
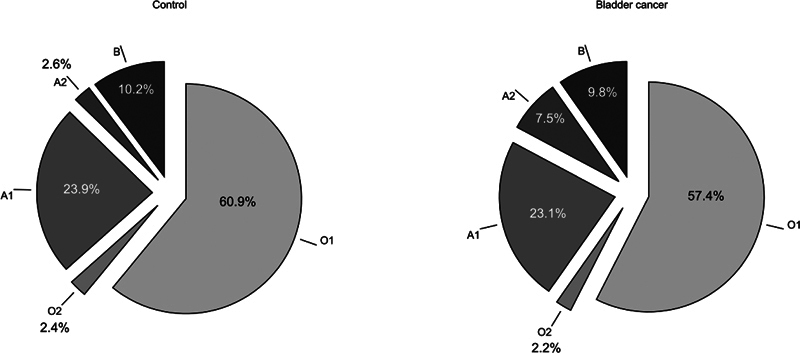
Distribution of ABO alleles in patients with bladder cancer and healthy controls.

A test of proportion has shown that a significantly lower proportion of patients with malignancy is present in patients with O1 ( *p* < 0.001), A1 ( *p* < 0.001), and B ( *p* = 0.013). In contrast, no statistical significance was present in patients with O2 ( *p* = 0.20) and A2 ( *p* = 0.468, Fig. 1 ).

**Fig. 2:**
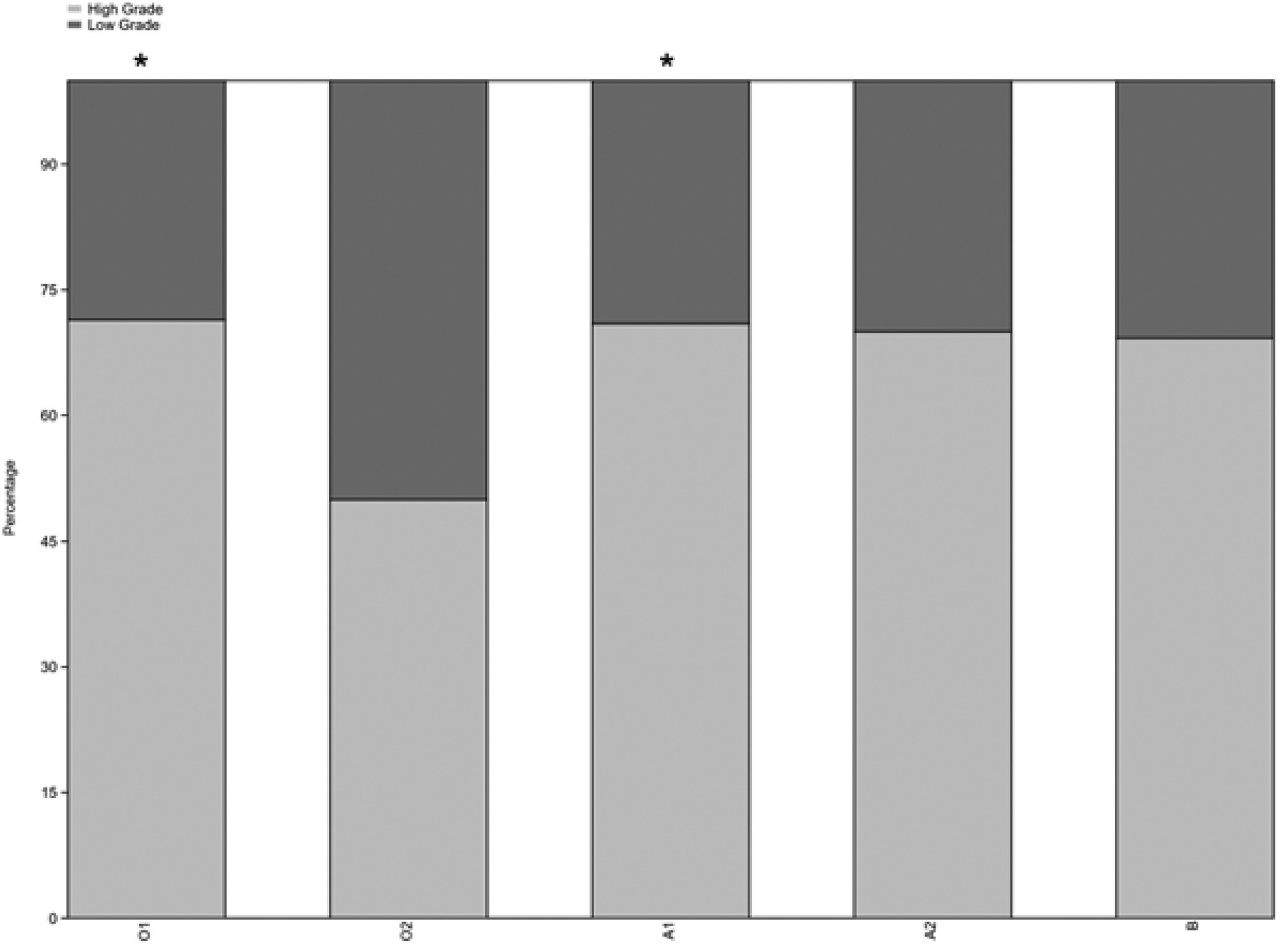
Allele distribution between high and low cancer grades.

In terms of tumor grade, no significant association was found between tumor grade (high and low) and alleles (χ ^2^ = 0.45, *p* = 0.978), whereas HG tumors are more frequent in patients with O1 (71.4%, *p* < 0.001) and A1 (70.1%, *p* = 0.019) alleles. No significant difference in tumor grade distribution was found in patients with O2, A2, and B alleles ( Fig. 2 ).

**Fig. 3:**
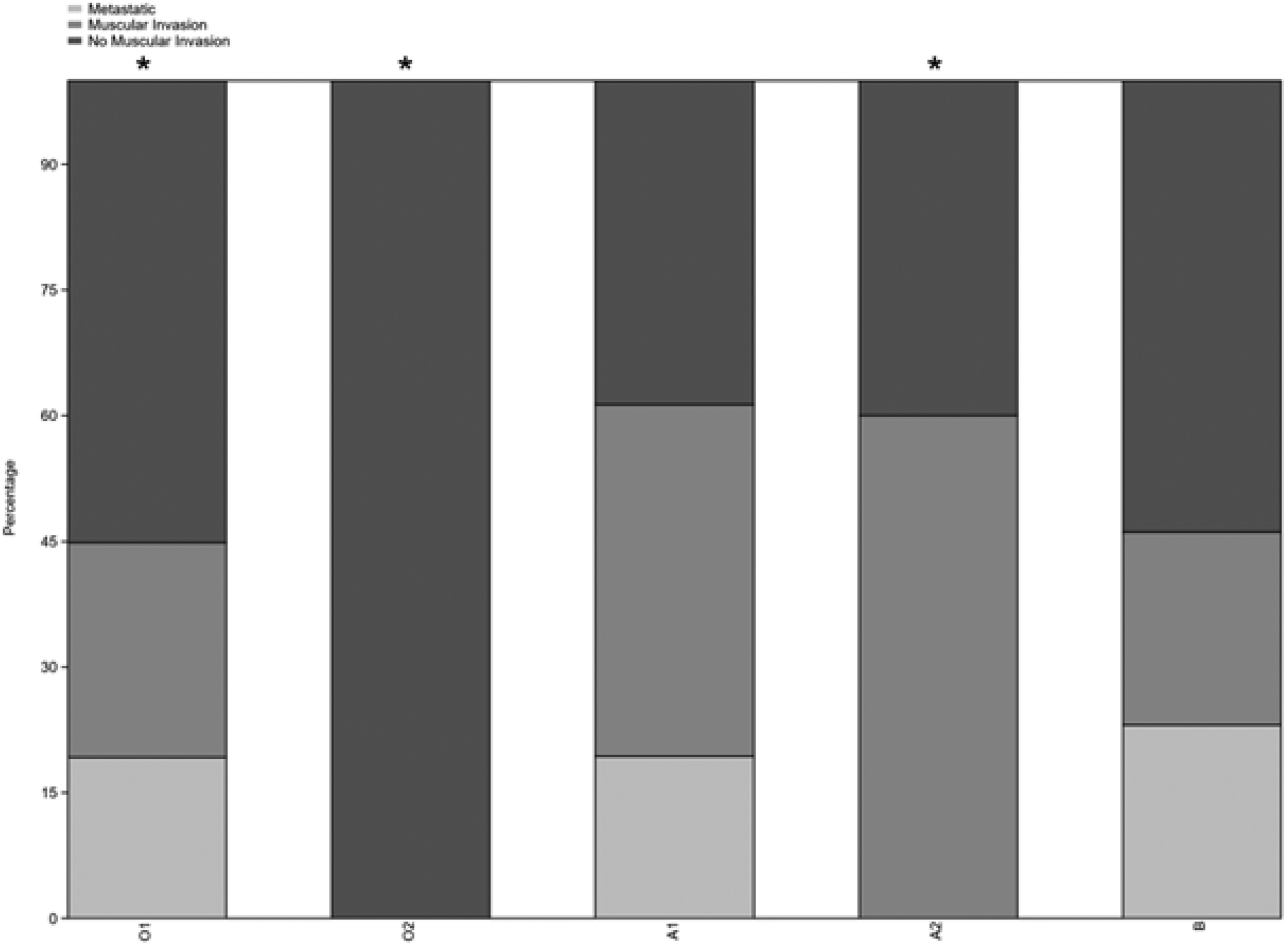
Allele distribution between nonmuscular invasive, muscular invasive, and metastatic cancer.

No association was found between tumor invasiveness (NMI, MI, M) and ABO alleles (χ ^2^ = 10.29, *p* = 0.245). NMI tumors were found to be in significantly higher proportion in patients with O1 (55.1%, *p* < 0.001) and O2 (100%, *p* = 0.045) alleles, whereas M tumors were in a significantly lower proportion in patients with A2 allele (0%, *p* = 0.024). No significant difference in the proportion of tumor invasiveness types was found in patients with A1 and B alleles ( Fig. 3 ).

In terms of tumor relapse, a significantly higher proportion of relapse was found in patients with O1 (70.2%, *p* < 0.001) and A1 (74.2%, *p* = 0.007) alleles. No significant difference was found in patients with O2, A2, and B alleles. A statistically significant result using a test of proportions strongly suggests that the proportion of one categorical variable differs significantly from the proportion of another categorical variable. In other words, it indicates that the observed difference in proportions is unlikely to have occurred due to random chance alone.

## Discussion

To analyze the potential of blood group as an adequate biomarker in the incidence of BC, distinguishing whether we type blood group immunohistochemically at the antigen level or genotyping at the allele level is necessary. Since the mid-20th century, the role of ABO in carcinogenesis has been intensively researched. The first research revealed that stomach cancer has been more frequent in bearers of the A blood group. 24 Based on the well-known method of determining a glycoprotein antigen of a blood group using serology, the hypotheses of pathogenetic mechanisms have also established that blood group antigens probably act as signaling molecules in the cell adhesion and cell recognition process. 25 Cartron et al determined that in addition to red blood cells, blood group antigens are also expressed on other tissues in various forms, resulting in wide functional diversity. 26 ABO is a system with the most immunogenic response in the human body, and based on the theory of avoiding immunologic responses, earlier research proved that for certain bladder tumors, a lack of A and B antigen expression on tumor cells when compared with the normal urothelium is a characteristic trait. 27 28 Beyond their role in immunogenic regulation, glycosyltransferase products have broad implications. Certain possible mechanisms analyzed for various tumors suggest that ABO antigens stimulate intracellular signaling in the direction of tumor angiogenesis or play a role in the inflammatory process by affecting the serum level of circulating inflammatory markers, soluble vascular endothelial growth factor for receptors 2 and 3, and soluble glycoprotein 130, thereby affecting the surveillance of malignant cells. 29 30 Srinivas et al found that BC patients with blood group A tend to have lower mortality and a more favorable histological cancer grade, whereas patients with blood group O have a worse histological type and higher mortality. 31 A study conducted on 494 patients with superficial BC indicated that blood group O carries the risk of disease recurrence and progression. 32 A meta-analysis from 2022 summarized 10 studies investigating the association between ABO system phenotypes and BC. An association with the treatment outcome was established in patients who had undergone radical cystectomy. Also discovered in patients with NMI carcinomas who had undergone TUR was that blood group A could affect the treatment outcome, whereas a lower incidence of disease recurrence and progression was found for blood group B. 33 According to a number of other authors, when bound on BC and ABO phenotype only, ABO antigens are not identified as appropriate biomarkers in BC outcome prognostication. 34 35

Our research did not find an association between ABO and BC at the phenotype (antigen) level. However, intriguing associations were found between specific blood group alleles and tumor grades, invasiveness, and relapse. We observed a lower risk as statistically significant in the appearance of BC among carriers O1, A1, and B alleles, whereas at the same time, a higher risk of HG and recurrent tumors in O1 and A1. We also determined the statistically significant lower proportion for M potential of A2 allele carriers and the statistically significant presence of superficial (NMI) tumors in carriers of noncoding O1 and O2 alleles.

As we already mentioned, blood group genotyping was performed using SNPs, which represent a genomic variant at a single base position of DNA. They are characteristic of a particular gene region, and in our research, we used them to identify alleles of the ABO blood system. In addition to determining a specific gene region, SNPs are also potential biomarkers in many cancer types. 36 SNP can occur in the coding region with or without a change in the amino acid. It can also occur in the regulatory gene region, where the result is a change in gene expression, or it can occur in the region between genes. 37 Therefore, their effect and pathogenetic mechanism on cancer susceptibility depends on the location of the SNPs. For example, SNPs in the promoter region decrease gene transcription by inhibiting transcription factor binding while promoting tumorigenesis in prostate, breast, colon, and pancreatic cancer or making an impact using epigenetic mechanisms through DNA methylation. 38 39

The classification of SNPs as exons is based on their ability to replace the encoded amino acid, i.e., nonsynonymous SNP change to the protein structure, and synonymous SNP alter protein structure indirectly by modulating mRNA stability. These changes affect cell signaling and levels of oncogenic and tumor suppressor proteins. 40 41

Research from 2009 identified 558,542 gene regions that may be potentially associated with the risk of developing pancreatic cancer, SNP rs 505922 located within the first intron of the ABO gene. It is called the protective allele (T), and its existence is evident in the linkage disequilibrium upon the deletion of the base pairs, which is responsible for noncoding the glycosyltransferase product of the O allele. It is among the first studies that has shown the link between the ABO system and cancer at the gene level. It also identified a significant association between SNPs, suggesting that the A and B antigens can be associated with an increased risk of pancreatic cancer, i.e., blood group O carries a lower risk of the disease. 42 Returning to the question from the beginning of the discussion, we can conclude from our results and literature that for the research on the link between the ABO system and the incidence of BC, blood group typing at the gene level is more appropriate than an immunohistochemical analysis at the antigen level. Blood groups are codominant, and we cannot determine the haplotype only from antigen data, where the specific genetic region, i.e., the allele and its constituent parts, pose a risk factor for the occurrence of cancer.

The ABO system is primarily involved in determining different blood types in individuals. It is not directly linked to carcinogenesis, but many studies have investigated potential associations over the last few decades. Although we have endeavored to explain our results using potential mechanisms published in previous research papers, it is important to note that they are not still fully understood. Is it a crucial role in interacting with the immune system, the expression of glycoconjugates or a variation of the gene region? Previous findings are inconsistent, and more research is needed to establish conclusive evidence regarding the specific mechanisms involved.

## Conclusion

This study provides an analysis of the association between ABO phenotype, genotype, and blood group alleles in patients with BC. While no significant differences were observed in ABO phenotype or genotype distribution, associations were found between specific blood group alleles and tumor grade, invasiveness, and relapse. These findings suggest a potential role for ABO alleles in BC progression and warrant further investigation with larger cohorts to validate these preliminary results.

The limitation of our study could possibly be the small number of samples. However, our study is among the first to assess the risk factors for BC at the level of ABO genotypes and alleles, which can provide more detailed information related to individual alleles as possible hereditary factors for the occurrence of BC. Understanding the genetic basis of BC can contribute to improving diagnostics, risk stratification, and targeted therapeutic interventions.
